# *N*-(4-Chloro-2-nitro­phen­yl)-5-methyl­isoxazole-4-carboxamide

**DOI:** 10.1107/S1600536811047994

**Published:** 2011-11-16

**Authors:** De-Cai Wang, Liang-Cheng Huang, Zhu-Yun Liu, Ping Wei, Ping-Kai Ou-yang

**Affiliations:** a State Key Laboratory of Materials-Oriented Chemical Engineering, School of Pharmaceutical Sciences, Nanjing University of Technology, Xinmofan Road No. 5 Nanjing, Nanjing 210009, People’s Republic of China.

## Abstract

In the title compound, C_11_H_8_ClN_3_O_4_, the dihedral angle between benzene and isoxazole rings is 9.92 (1) °. The nitro group is almost coplanar with the benzene ring with an O—N—C—C torsion angle of 8.4 (3)°. The mol­ecular conformation is stabilized by an intra­molecular N—H⋯O hydrogen bond, closing a six-membered ring.

## Related literature

For applications of leflunomide [systematic name: 5-methyl-*N*-[4-(trifluoro­meth­yl) phen­yl]-isoxazole-4-carboxamide] in the treatment of rheumatoid arthritis, see: Shaw *et al.* (2011 ▶); Schattenkirchner (2000 ▶). The title compound was synthesized as an immunomodulating leflunomide analog; for another immunomodulating leflunomide analog, see: Huang *et al.* (2003 ▶).
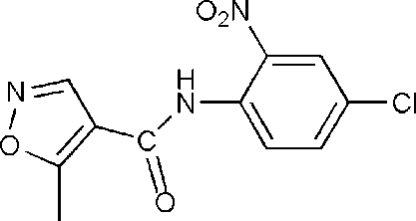


## Experimental

### 

#### Crystal data

C_11_H_8_ClN_3_O_4_
*M* *_r_* = 281.65
Monoclinic, 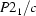

*a* = 5.3870 (11) Å
*b* = 23.537 (5) Å
*c* = 9.4600 (19) Å
β = 99.86 (3)°
*V* = 1181.8 (4) Å^3^
*Z* = 4
Mo *K*α radiation
μ = 0.34 mm^−1^
*T* = 293 K
0.30 × 0.20 × 0.10 mm

#### Data collection

Enraf–Nonius CAD-4 diffractometer
Absorption correction: ψ scan (North *et al.*, 1968 ▶) *T*_min_ = 0.905, *T*_max_ = 0.967
4645 measured reflections
2121 independent reflections
1558 reflections with *I* > 2σ(*I*)
*R*_int_ = 0.044
3 standard reflections every 200 reflections  intensity decay: 1%

#### Refinement

*R*[*F* ^2^ > 2σ(*F* ^2^)] = 0.042
*wR*(*F* ^2^) = 0.143
*S* = 1.02
2121 reflections
174 parameters
H-atom parameters constrained
Δρ_max_ = 0.18 e Å^−3^
Δρ_min_ = −0.22 e Å^−3^

### 

Data collection: *CAD-4 EXPRESS* (Enraf–Nonius, 1994 ▶); cell refinement: *CAD-4 EXPRESS*; data reduction: *XCAD4* (Harms & Wocadlo, 1995 ▶); program(s) used to solve structure: *SHELXS97* (Sheldrick, 2008 ▶); program(s) used to refine structure: *SHELXL97* (Sheldrick, 2008 ▶); molecular graphics: *SHELXTL* (Sheldrick, 2008 ▶); software used to prepare material for publication: *SHELXTL*.

## Supplementary Material

Crystal structure: contains datablock(s) I, global. DOI: 10.1107/S1600536811047994/ld2035sup1.cif

Structure factors: contains datablock(s) I. DOI: 10.1107/S1600536811047994/ld2035Isup2.hkl

Additional supplementary materials:  crystallographic information; 3D view; checkCIF report

## Figures and Tables

**Table 1:** Hydrogen-bond geometry (Å, °)

*D*—H⋯*A*	*D*—H	H⋯*A*	*D*⋯*A*	*D*—H⋯*A*
N2—H2*B*⋯O2	0.86	1.94	2.629 (3)	136

**Fig. 1.**
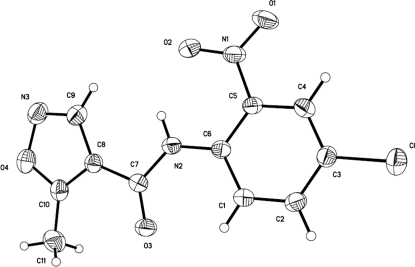
The structure of the title compound, showing the atomic numbering scheme. Non-H atoms are shown with 30% probability displacement ellipsoids.

## supplementary crystallographic information

### Comment

Leflunomide is one of the most effective isoxazole-containing disease-modifying drugs for treating rheumatoid arthritis(Shaw *et al.*, 2011; Schattenkirchner, 2000). Many leflunomide analogs have been synthesized and exhibit potent immunomodulating effect (Huang, *et al.*, 2003). The title compound, 5-methyl-*N*-(4-chloro-2-nitrophenyl)isoxazole-4-carboxamide (I), was synthesized as a novel and potent immunomodulating leflunomide analog. We report herein its crystal structure. The molecular structure of the title compound is shown in Fig. 1. The nitro group is approximately coplanar with the benzene ring, as indicated by the torsion angle O1—N1—C5—C4 of 8.4 (3)°. The amide group is also coplanar with the benzene and isoxazole rings [torsion angles N2—C7—C8—C9 and C7—N2—C6—C1 are -8.9 (4) and -3.1 (4)°], respectively. The molecular conformation is stabilized by an intra-molecular N—H···O hydrogen bond (Table 1). In the crystal, infinite zigzag chain are formed via short inter-molecular Cl···O contacts of 3.089 (3) Å.

### Experimental

A solution of 0.05 mol of 5-methylisoxazole-4-carboxylic acid chloride (7.3 g) in 20 ml of acetonitrile was added dropwise, while stirring, to 0.1 mol of 4-chloro-2-nitroaniline (17.2 g), dissolved in 150 ml of acetonitrile, at room temperature. After stirring for 40 more minutes, the precipitated 4-chloro-2-nitroaniline hydrochloride was filtered off and washed with 100 ml portions of acetonitrile, and the combined filtrates were concentrated under reduced pressure. 9.6 g (65% of theory) of yellow crytalline 5-methyl-*N*-(4-Chloro-2-nitrophenyl)isoxazole-4-carboxamide were thus obtained. Crystals of (I) suitable for X-ray diffraction were obtained by slow evaporation of toluene solution.

### Refinement

Carbon- and nitrogen-bound H atoms were placed in calculated positions and were treated as riding on the parent C or N atoms with C—H = 0.96 (methyl), 0.97 (methylene) and N—H = 0.86 Å, *U*_iso_(H) = 1.2 *U*_eq_(C, N). The positions of methyl hydrogens were optimized rotationally with *U*_iso_(H) = 1.5 *U*_eq_(C).

### Crystal data

**Table:**

C_11_H_8_ClN_3_O_4_	*F*(000) = 576
*M**_r_* = 281.65	*D*_x_ = 1.583 Mg m^−^^3^
Monoclinic, *P*2_1_/*c*	Mo *K*α radiation, λ = 0.71073 Å
Hall symbol: -P 2ybc	Cell parameters from 25 reflections
*a* = 5.3870 (11) Å	θ = 9–13°
*b* = 23.537 (5) Å	µ = 0.34 mm^−^^1^
*c* = 9.4600 (19) Å	*T* = 293 K
β = 99.86 (3)°	Block, white
*V* = 1181.8 (4) Å^3^	0.30 × 0.20 × 0.10 mm
*Z* = 4	

### Data collection

**Table:**

Enraf–Nonius CAD-4 diffractometer	1558 reflections with *I* > 2σ(*I*)
Radiation source: fine-focus sealed tube	*R*_int_ = 0.044
graphite	θ_max_ = 25.2°, θ_min_ = 1.7°
ω/2θ scans	*h* = 0→6
Absorption correction: ψ scan (North *et al.*, 1968)	*k* = −28→28
*T*_min_ = 0.905, *T*_max_ = 0.967	*l* = −11→11
4645 measured reflections	3 standard reflections every 200 reflections
2121 independent reflections	intensity decay: 1%

### Refinement

**Table:**

Refinement on *F*^2^	Secondary atom site location: difference Fourier map
Least-squares matrix: full	Hydrogen site location: inferred from neighbouring sites
*R*[*F*^2^ > 2σ(*F*^2^)] = 0.042	H-atom parameters constrained
*wR*(*F*^2^) = 0.143	*w* = 1/[σ^2^(*F*_o_^2^) + (0.090*P*)^2^] where *P* = (*F*_o_^2^ + 2*F*_c_^2^)/3
*S* = 1.02	(Δ/σ)_max_ < 0.001
2121 reflections	Δρ_max_ = 0.18 e Å^−^^3^
174 parameters	Δρ_min_ = −0.22 e Å^−^^3^
0 restraints	Extinction correction: *SHELXL*, Fc^*^=kFc[1+0.001xFc^2^λ^3^/sin(2θ)]^-1/4^
Primary atom site location: structure-invariant direct methods	Extinction coefficient: 0.010 (3)

### Special details

**Table:**

Geometry. All e.s.d.'s (except the e.s.d. in the dihedral angle between two l.s. planes) are estimated using the full covariance matrix. The cell e.s.d.'s are taken into account individually in the estimation of e.s.d.'s in distances, angles and torsion angles; correlations between e.s.d.'s in cell parameters are only used when they are defined by crystal symmetry. An approximate (isotropic) treatment of cell e.s.d.'s is used for estimating e.s.d.'s involving l.s. planes.
Refinement. Refinement of *F*^2^ against ALL reflections. The weighted *R*-factor *wR* and goodness of fit *S* are based on *F*^2^, conventional *R*-factors *R* are based on *F*, with *F* set to zero for negative *F*^2^. The threshold expression of *F*^2^ > σ(*F*^2^) is used only for calculating *R*-factors(gt) *etc*. and is not relevant to the choice of reflections for refinement. *R*-factors based on *F*^2^ are statistically about twice as large as those based on *F*, and *R*- factors based on ALL data will be even larger.

### Fractional atomic coordinates and isotropic or equivalent isotropic displacement parameters (Å^2^)

**Table:**

	*x*	*y*	*z*	*U*_iso_*/*U*_eq_	
Cl	1.14810 (15)	0.27507 (3)	0.51209 (9)	0.0712 (3)	
N1	0.5249 (4)	0.33771 (10)	0.8313 (2)	0.0534 (6)	
C1	0.8613 (5)	0.42551 (11)	0.5906 (3)	0.0476 (6)	
H1A	0.8768	0.4628	0.5606	0.057*	
O1	0.5463 (5)	0.28905 (9)	0.8742 (3)	0.0868 (8)	
O2	0.3751 (4)	0.37087 (9)	0.8716 (2)	0.0611 (5)	
C2	0.9988 (5)	0.38350 (11)	0.5390 (3)	0.0507 (6)	
H2A	1.1070	0.3927	0.4757	0.061*	
N2	0.5564 (4)	0.45566 (9)	0.7398 (2)	0.0469 (5)	
H2B	0.4559	0.4440	0.7951	0.056*	
N3	0.0876 (5)	0.56302 (12)	0.9317 (3)	0.0656 (7)	
O3	0.6784 (4)	0.53680 (8)	0.6379 (2)	0.0586 (5)	
C3	0.9774 (5)	0.32777 (11)	0.5806 (3)	0.0475 (6)	
O4	0.1878 (4)	0.61421 (9)	0.8857 (2)	0.0658 (6)	
C4	0.8225 (5)	0.31427 (11)	0.6765 (3)	0.0501 (6)	
H4A	0.8099	0.2768	0.7060	0.060*	
C5	0.6850 (5)	0.35645 (10)	0.7294 (2)	0.0434 (6)	
C6	0.6978 (4)	0.41348 (10)	0.6875 (2)	0.0406 (5)	
C7	0.5541 (5)	0.51330 (10)	0.7158 (2)	0.0433 (6)	
C8	0.3842 (4)	0.54381 (11)	0.7965 (2)	0.0444 (6)	
C9	0.2056 (5)	0.52340 (12)	0.8774 (3)	0.0555 (7)	
H9A	0.1763	0.4850	0.8901	0.067*	
C10	0.3644 (5)	0.60153 (11)	0.8058 (3)	0.0522 (7)	
C11	0.4990 (7)	0.65000 (12)	0.7545 (4)	0.0731 (9)	
H11A	0.4181	0.6848	0.7737	0.110*	
H11B	0.4958	0.6464	0.6532	0.110*	
H11C	0.6705	0.6503	0.8035	0.110*	

### Atomic displacement parameters (Å^2^)

**Table:**

	*U*^11^	*U*^22^	*U*^33^	*U*^12^	*U*^13^	*U*^23^
Cl	0.0730 (5)	0.0583 (5)	0.0884 (6)	0.0099 (4)	0.0308 (4)	−0.0103 (4)
N1	0.0613 (14)	0.0467 (13)	0.0561 (12)	−0.0111 (11)	0.0211 (11)	0.0031 (10)
C1	0.0504 (14)	0.0448 (14)	0.0502 (13)	−0.0018 (12)	0.0158 (11)	0.0077 (11)
O1	0.121 (2)	0.0491 (13)	0.1062 (18)	−0.0060 (12)	0.0648 (16)	0.0174 (11)
O2	0.0612 (12)	0.0607 (12)	0.0687 (12)	−0.0006 (10)	0.0320 (10)	0.0094 (9)
C2	0.0515 (15)	0.0515 (16)	0.0530 (14)	−0.0043 (12)	0.0199 (12)	0.0013 (11)
N2	0.0524 (12)	0.0432 (12)	0.0493 (11)	−0.0006 (9)	0.0210 (10)	0.0049 (9)
N3	0.0568 (14)	0.0717 (18)	0.0722 (15)	0.0055 (12)	0.0221 (12)	−0.0037 (12)
O3	0.0713 (13)	0.0431 (11)	0.0691 (11)	−0.0025 (9)	0.0335 (10)	0.0048 (8)
C3	0.0449 (13)	0.0467 (15)	0.0520 (14)	0.0003 (11)	0.0112 (11)	−0.0044 (11)
O4	0.0620 (12)	0.0666 (14)	0.0703 (12)	0.0135 (10)	0.0152 (10)	−0.0107 (10)
C4	0.0541 (15)	0.0379 (14)	0.0587 (15)	−0.0039 (11)	0.0107 (13)	0.0027 (11)
C5	0.0434 (13)	0.0429 (14)	0.0454 (13)	−0.0068 (11)	0.0123 (11)	0.0015 (10)
C6	0.0430 (13)	0.0411 (13)	0.0377 (11)	−0.0023 (10)	0.0069 (10)	0.0009 (9)
C7	0.0459 (13)	0.0408 (13)	0.0431 (13)	−0.0014 (11)	0.0069 (11)	0.0017 (10)
C8	0.0433 (13)	0.0464 (15)	0.0428 (12)	0.0017 (11)	0.0053 (10)	−0.0003 (10)
C9	0.0513 (15)	0.0590 (18)	0.0577 (15)	−0.0006 (13)	0.0140 (13)	−0.0033 (13)
C10	0.0521 (15)	0.0536 (16)	0.0506 (14)	0.0088 (13)	0.0080 (12)	−0.0030 (12)
C11	0.089 (2)	0.0471 (17)	0.086 (2)	0.0019 (16)	0.0222 (18)	−0.0016 (15)

### Geometric parameters (Å, °)

**Table:**

Cl—C3	1.734 (3)	O3—C7	1.210 (3)
N1—O1	1.214 (3)	C3—C4	1.371 (4)
N1—O2	1.230 (3)	O4—C10	1.346 (3)
N1—C5	1.467 (3)	C4—C5	1.382 (3)
C1—C2	1.374 (4)	C4—H4A	0.9300
C1—C6	1.405 (3)	C5—C6	1.405 (3)
C1—H1A	0.9300	C7—C8	1.475 (3)
C2—C3	1.380 (4)	C8—C10	1.367 (4)
C2—H2A	0.9300	C8—C9	1.412 (4)
N2—C7	1.375 (3)	C9—H9A	0.9300
N2—C6	1.393 (3)	C10—C11	1.477 (4)
N2—H2B	0.8600	C11—H11A	0.9600
N3—C9	1.285 (3)	C11—H11B	0.9600
N3—O4	1.419 (3)	C11—H11C	0.9600
			
O1—N1—O2	121.7 (2)	C6—C5—N1	122.4 (2)
O1—N1—C5	118.0 (2)	N2—C6—C1	122.0 (2)
O2—N1—C5	120.3 (2)	N2—C6—C5	121.6 (2)
C2—C1—C6	121.5 (2)	C1—C6—C5	116.3 (2)
C2—C1—H1A	119.2	O3—C7—N2	124.1 (2)
C6—C1—H1A	119.2	O3—C7—C8	123.3 (2)
C1—C2—C3	120.4 (2)	N2—C7—C8	112.5 (2)
C1—C2—H2A	119.8	C10—C8—C9	103.6 (2)
C3—C2—H2A	119.8	C10—C8—C7	125.4 (2)
C7—N2—C6	129.4 (2)	C9—C8—C7	131.0 (2)
C7—N2—H2B	115.3	N3—C9—C8	113.6 (3)
C6—N2—H2B	115.3	N3—C9—H9A	123.2
C9—N3—O4	104.7 (2)	C8—C9—H9A	123.2
C4—C3—C2	119.9 (2)	O4—C10—C8	109.1 (2)
C4—C3—Cl	120.2 (2)	O4—C10—C11	116.5 (3)
C2—C3—Cl	119.8 (2)	C8—C10—C11	134.3 (3)
C10—O4—N3	109.1 (2)	C10—C11—H11A	109.5
C3—C4—C5	119.8 (2)	C10—C11—H11B	109.5
C3—C4—H4A	120.1	H11A—C11—H11B	109.5
C5—C4—H4A	120.1	C10—C11—H11C	109.5
C4—C5—C6	121.9 (2)	H11A—C11—H11C	109.5
C4—C5—N1	115.7 (2)	H11B—C11—H11C	109.5
			
C6—C1—C2—C3	0.7 (4)	C4—C5—C6—C1	−1.0 (3)
C1—C2—C3—C4	−1.5 (4)	N1—C5—C6—C1	179.2 (2)
C1—C2—C3—Cl	178.87 (19)	C6—N2—C7—O3	2.0 (4)
C9—N3—O4—C10	−0.4 (3)	C6—N2—C7—C8	−177.5 (2)
C2—C3—C4—C5	1.1 (4)	O3—C7—C8—C10	−7.0 (4)
Cl—C3—C4—C5	−179.31 (19)	N2—C7—C8—C10	172.5 (2)
C3—C4—C5—C6	0.2 (4)	O3—C7—C8—C9	171.5 (2)
C3—C4—C5—N1	180.0 (2)	N2—C7—C8—C9	−8.9 (4)
O1—N1—C5—C4	8.4 (3)	O4—N3—C9—C8	0.4 (3)
O2—N1—C5—C4	−171.8 (2)	C10—C8—C9—N3	−0.2 (3)
O1—N1—C5—C6	−171.8 (2)	C7—C8—C9—N3	−179.0 (2)
O2—N1—C5—C6	7.9 (4)	N3—O4—C10—C8	0.3 (3)
C7—N2—C6—C1	−3.1 (4)	N3—O4—C10—C11	−177.0 (2)
C7—N2—C6—C5	176.7 (2)	C9—C8—C10—O4	−0.1 (3)
C2—C1—C6—N2	−179.6 (2)	C7—C8—C10—O4	178.8 (2)
C2—C1—C6—C5	0.6 (3)	C9—C8—C10—C11	176.5 (3)
C4—C5—C6—N2	179.2 (2)	C7—C8—C10—C11	−4.6 (5)
N1—C5—C6—N2	−0.6 (4)		

### Hydrogen-bond geometry (Å, °)

**Table:**

*D*—H···*A*	*D*—H	H···*A*	*D*···*A*	*D*—H···*A*
N2—H2B···O2	0.86	1.94	2.629 (3)	136.
